# Subtype-dependent prognostic significance of Ki-67, histological grade, and vascular invasion in breast and colorectal cancer

**DOI:** 10.1097/MD.0000000000050646

**Published:** 2026-09-11

**Authors:** Hongyan Ji, Binzheng Liu, Lisha Xie

**Affiliations:** a Jiande First People’s Hospital, Jiande, Zhejiang, China.

**Keywords:** breast cancer, colorectal cancer, Ki-67, mismatch repair, molecular subtype, prognosis, vascular invasion

## Abstract

Traditional pathological markers remain relevant in prognostic assessment for solid tumors, but their clinical value may differ according to tumor biological context. This study evaluated the prognostic significance of Ki-67, histological grade, and vascular invasion (VI) in breast and colorectal cancer, with emphasis on subtype-specific differences. This single-center retrospective cohort included 846 patients with breast cancer or colorectal cancer who underwent curative-intent surgery between 2015 and 2022. Ki-67 expression, histological grade, and VI were extracted from pathological records. The pooled analysis was designed as an exploratory cross-cancer comparison of shared pathological features rather than as an assumption of biological equivalence between the 2 malignancies; cancer-specific and molecular-context analyses were therefore emphasized. Overall survival was assessed using Cox proportional hazards regression, with subgroup analyses according to breast cancer surrogate molecular subtype and colorectal cancer mismatch repair status. A simple Ki-67/VI score was explored for risk stratification. In multivariable analysis, increased age, stage III disease, high Ki-67 expression, and VI were independently associated with worse overall survival, whereas histological grade was not retained as an independent predictor. In breast cancer, the prognostic effect of Ki-67 was evident in luminal B and triple-negative subtypes but not in luminal A disease. In colorectal cancer, VI showed stronger prognostic value in proficient mismatch repair tumors than in deficient mismatch repair tumors. The combined Ki-67/VI score provided incremental prognostic stratification and showed better discriminatory performance than any single marker alone. Ki-67 and VI were associated with prognosis in this retrospective cohort, and the magnitude of these associations varied according to tumor biological context. The Ki-67/VI score should be regarded as exploratory and hypothesis-generating pending internal optimism correction and external validation. Interpretation of conventional pathological indicators should therefore remain cancer- and subtype-specific.

## 1. Introduction

Breast cancer and colorectal cancer are common malignancies that pose a serious threat to human health worldwide, with both incidence and mortality ranking among the highest. Although modern oncology has made significant progress in diagnosis and treatment, particularly in precision treatment strategies based on molecular classification, the inter-individual heterogeneity in patient prognosis remains a major clinical challenge.^[1]^ Accurate prognostic assessment is fundamental for developing individualized treatment plans, optimizing the allocation of medical resources, and improving long-term patient survival outcomes. Currently, the internationally adopted tumor-node-metastasis (TNM) staging system provides a basic framework for prognostic judgment; however, its focus on anatomical extent makes it difficult to fully characterize the intrinsic biological heterogeneity of tumors.^[2]^ For instance, within stage II breast cancer, the clinical outcomes of luminal A and triple-negative patients differ markedly; similarly, among stage III colorectal cancer patients, those with proficient mismatch repair (pMMR) and those with deficient mismatch repair (dMMR) show significant differences in their response to adjuvant therapy and long-term survival.^[3]^ Therefore, moving beyond traditional anatomical staging to integrate pathological indicators that more directly reflect malignant biological behavior has become an important research direction for enhancing the precision of prognostic assessment.

Among various pathological indicators, the tumor cell proliferation index (Ki-67), histological grade, and vascular invasion (VI) status are garnering increasing attention. Ki-67, a marker of cellular proliferative activity, exhibits expression levels closely associated with cell cycle progression.^[4]^ Research by Uxa et al^[5]^ elucidated the gene expression regulatory mechanisms of Ki-67, establishing the theoretical foundation for its role as a proliferation marker. In clinical studies, Choi et al^[6]^ investigated the impact of Ki-67 assessment based on biopsy versus surgical specimens on breast cancer prognosis, while the Viale team clarified the pivotal role of Ki-67 in distinguishing between luminal A and luminal B breast cancer subtypes. In the field of colorectal cancer, early work by Ishida et al^[7]^ suggested that high Ki-67 expression is associated with poor prognosis in Dukes stage C patients. However, whether the prognostic value of Ki-67 remains consistent across different molecular subtype contexts is still a subject of debate in the current literature, warranting further validation.

Histological grade is a classical indicator for assessing tumor differentiation and malignancy. Through a study on multi-gene mutation signatures, Zhuang et al^[8]^ confirmed the association of pathological grade with diagnostic staging and prognosis in colorectal cancer. In breast cancer, the Nottingham histological grading system has been widely recognized as an independent prognostic factor; recently, Freitas et al^[9]^ further explored its biological link to patient outcomes via gene expression profiling analysis. Although grading systems hold significant value, their assessment involves a degree of subjectivity, and their standards and implications across different cancer types require nuanced interpretation in conjunction with molecular context.

Vascular invasion represents a critical step in tumor dissemination. Prior studies support its prognostic relevance in both breast and colorectal cancer, although definitions and effect sizes vary across settings. Most published studies evaluate 1 marker within 1 cancer type, whereas the present study was conceived as an exploratory cross-cancer comparison of 3 routinely reported pathological features – Ki-67, histological grade, and VI – in 2 common epithelial malignancies for which these variables are routinely available. Breast and colorectal cancers were not combined on the premise that they share a common baseline hazard, treatment pathway, or tumor biology. Rather, the pooled cohort was used to examine whether broad directional associations of shared pathological features could be observed across cancers, while cancer-specific molecular-context analyses (breast surrogate subtype and colorectal MMR status) were used to investigate heterogeneity. Accordingly, pooled estimates are interpreted as descriptive/exploratory, and disease-specific findings are given greater biological weight.

## 2. Materials and methods

### 2.1. Study population

This study was approved by the Ethics Committee of Jiande First People’s Hospital. We conducted a retrospective cohort analysis using data extracted from the pathology archives and linked electronic health records of a tertiary-care cancer center. Consecutive breast and colorectal cancer patients who underwent 1st-time curative-intent radical surgery at our institution between January 1, 2015, and December 31, 2022, were included.

### 2.2. Inclusion and exclusion criteria

Inclusion criteria: First, pathological confirmation via postoperative paraffin-embedded sections of primary invasive breast carcinoma (according to WHO classification, excluding ductal carcinoma in situ and lobular carcinoma in situ) or primary colorectal adenocarcinoma (excluding neuroendocrine tumors, stromal tumors, etc). Second, postoperative pathology reports must contain clear records available for analysis: Ki-67 proliferation index (percentage of positive cells), pathological grade (Nottingham histological grade system for breast cancer; differentiation degree recorded for colorectal cancer), and VI status (present or absent). Third, possession of complete pre and postoperative clinical data and availability of follow-up information suitable for survival analysis (including at least 1 postoperative review record or a confirmed date of death).

Exclusion criteria: First, preoperative imaging or postoperative pathology confirming distant metastasis (M1 stage). Second, history of other malignant tumors within the past 5 years, excluding basal cell carcinoma of the skin or cervical carcinoma in situ. Third, receipt of neoadjuvant chemotherapy, radiotherapy, targeted therapy, or endocrine therapy prior to surgery. Fourth, perioperative (within 30 days post-surgery) death due to non-oncological causes such as surgical complications. Fifth, missing or ambiguous data for key pathological indicators (Ki-67, grade, and VI) or survival follow-up information, rendering them uninterpretable.

### 2.3. Sample size estimation

This was an exploratory retrospective analysis; sample size estimation was primarily based on feasibility and the requirements for subgroup analysis. Estimation was performed using GPower 3.1 software (Heinrich-Heine-Universität Düsseldorf). With a significance level (α) of 0.05, statistical power (1 − β) of 0.80, an expected medium effect size (hazard ratio, HR ≈ 1.5), and consideration of approximately a 15% loss to follow-up rate, the estimated required total sample size was approximately 800 cases. The final plan was to include 800 to 1000 patients, with a breast-to-colorectal cancer ratio of approximately 1:1.2 to 1:1.5, to ensure that each subgroup in subsequent analyses based on molecular subtype (e.g., 4 breast cancer subtypes, pMMR/dMMR status in colorectal cancer) would have a basic sample size for preliminary statistical comparison.

Ki-67 was recorded as the percentage of positively stained tumor-cell nuclei. For breast cancer, <14% versus ≥14% was retained for categorical analyses to preserve consistency with the historical pathology dataset; because Ki-67 is intrinsically continuous and assay-dependent, this threshold was not interpreted as a universal biological boundary. For colorectal cancer, 20% was treated as a prespecified, literature-informed threshold for categorical presentation rather than an outcome-optimized cutoff derived and validated in the same cohort. Sensitivity analyses examined alternative cutoffs of 15%, 25%, and 30%, and Ki-67 was additionally modeled continuously and with restricted cubic splines (RCSs).

### 2.4. Grouping criteria

Ki-67 was recorded as the percentage of positively stained tumor-cell nuclei. For breast cancer, <14% versus ≥14% was retained for categorical analyses to preserve consistency with the threshold used in the original pathology dataset and earlier surrogate-classification literature; however, because Ki-67 is intrinsically continuous and assay-dependent, categorical results were not interpreted as evidence of a universal biological threshold. For colorectal cancer, 20% was used as a prespecified, literature-informed threshold for categorical presentation. Therefore, continuous-variable analyses and RCS sensitivity analyses are specified below as the preferred assessment of dose–response and nonlinearity.

Breast cancer molecular subtypes were defined using immunohistochemical surrogate categories. Luminal A-like tumors were estrogen receptor (ER)-positive, human epidermal growth factor receptor 2 (HER2)-negative, progesterone receptor (PR) ≥20%, with low Ki-67; luminal B-like/HER2-negative tumors were ER-positive, HER2-negative with either PR <20% and/or high Ki-67; luminal B-like/HER2-positive tumors were ER-positive and HER2-positive. HER2-positive/non-luminal tumors were ER/PR-negative and HER2-positive, and triple-negative tumors were ER-, PR-, and HER2-negative. Because Ki-67 contributes to luminal A-like versus luminal B-like classification, this creates a potential incorporation bias when evaluating Ki-67 prognosis within those subtypes. Therefore, a sensitivity analysis using receptor/HER2-defined strata independent of Ki-67 was specified.

VI was recorded as present or absent. All equivocal cases were reviewed independently by 2 senior pathologists blinded to clinical outcomes, with consensus reached through discussion.

Subgroup analyses were predefined based on established molecular classifications. Breast cancer cases were categorized into molecular subtypes (luminal A, luminal B, HER2-overexpressing, and triple-negative) according to ER, PR, and HER2 status. Colorectal cancer cases were stratified by MMR status into pMMR, and dMMR groups based on immunohistochemical expression of MLH1, PMS2, MSH2, and MSH6.

### 2.5. Data collection and quality control

Data were extracted from electronic medical records and pathological archives using a standardized case report form. To ensure accuracy, dual independent data entry with cross-verification was performed. Two researchers independently reviewed the records for eligibility and data extraction; discrepancies were resolved by consensus or arbitration by a 3rd investigator. The collected variables included:

Demographic and baseline clinical characteristics: Age, sex, body mass index, primary tumor location, preoperative serum tumor markers (carcinoembryonic antigen, carbohydrate antigen 15-3, and carbohydrate antigen 19-9), and clinical TNM stage assessed by imaging.
Core pathological data: Pathological staging adhered to the 8th edition of the American Joint Committee on Cancer Staging Manual. All histopathological indicators – including Ki-67 index, Nottingham grade (breast) or differentiation grade (colorectal), VI (specifying lymphatic or blood vessel involvement), perineural invasion, and resection margin status – were confirmed via centralized rereview by 2 experienced pathologists unaware of patient outcomes.
Cancer-specific biomarkers: For breast cancer, histological type, ER/PR expression levels, and HER2 status (by immunohistochemistry and fluorescence in situ hybridization where indicated) were recorded. For colorectal cancer, histological type and MMR protein expression (MLH1, PMS2, MSH2, and MSH6) were documented.
Treatment details: Surgical date and procedure and postoperative adjuvant treatment were collected, including chemotherapy, radiotherapy, anti-HER2 targeted therapy, and endocrine therapy where applicable. Because postoperative treatment is strongly determined by cancer type, stage, receptor/MMR profile, age, and pathological risk features, treatment can act both as a prognostic factor and as a source of confounding by indication. The primary pathological association models therefore require disease-specific treatment sensitivity analyses rather than a single pooled “treatment” variable. For breast cancer, broad indicators of adjuvant chemotherapy, radiotherapy, endocrine therapy, and anti-HER2 therapy should be considered as appropriate; for colorectal cancer, adjuvant chemotherapy should be considered according to stage and regimen availability.
Follow-up data: Patients were followed via outpatient visits, telephone interviews, and linkage with vital statistics registries. Dates and evidence (imaging or pathology) for overall survival (OS) and disease-free survival (DFS) events – including local recurrence, regional nodal metastasis, distant metastasis, or death from any cause – were recorded. Follow-up was censored on June 30, 2025.

### 2.6. Observation endpoints and definitions

Primary endpoint: OS, defined as the time from the date of surgery to death from any cause. Surviving patients were censored at the date of last follow-up.

Secondary endpoint: DFS, defined as the time from the date of surgery to the 1st occurrence of any of the following events: confirmed local recurrence, regional lymph node metastasis, distant metastasis, or death from any cause. An elevation in tumor markers alone without imaging or pathological confirmation was not considered an event.

### 2.7. Statistical analysis

Statistical analyses were performed with SPSS 26.0 (IBM Corp.) and R 4.2.1 (R Foundation). Statistical significance was defined by a 2-tailed *P* value threshold of <.05. Descriptive data are summarized as follows: continuous variables with normal distribution are expressed as mean ± standard deviation and compared using Student *t* test; non-normally distributed continuous variables are presented as median (interquartile range) and compared with the Mann–Whitney *U* or Kruskal–Wallis test; categorical variables are reported as frequencies (percentages) and analyzed using the χ^2^ test or Fisher exact test, as appropriate. For time-to-event endpoints, survival probabilities were estimated with the Kaplan–Meier method, and differences between groups were assessed via the log-rank test. Factors associated with OS and DFS were identified using univariate and multivariate Cox proportional hazards regression models. Results are reported as HRs with corresponding 95% confidence intervals (CIs). Variables showing a univariate *P* value <.1 or considered clinically relevant were included in the subsequent multivariate model.

The ability of individual and combined pathological markers to discriminate 5-year OS status (alive vs deceased) was evaluated using the receiver operating characteristic (ROC) analyses specified in the original study. The area under the curve (AUC) and its 95% CI were calculated for each marker – Ki-67 (as a continuous variable for ROC analysis), histological grade (ordinal), and VI (binary) – as well as for 2 integrated exploratory models: a simple score (0–2 points, assigning 1 point each for high Ki-67 expression and presence of VI) and a multivariable logistic regression model incorporating all 3 markers. Comparisons of AUC values were conducted with DeLong test. No additional bootstrap correction, time-dependent AUC, calibration analysis, decision-curve analysis, or RCS modeling was added in the present revision. Accordingly, the combined score is described throughout as exploratory rather than as a validated clinical prediction model.

Subgroup analyses were conducted within the recorded breast cancer molecular subtypes and colorectal cancer MMR status groups using the analyses prespecified in the original study. These subgroup findings were interpreted cautiously as exploratory evidence of possible heterogeneity rather than as definitive proof of effect modification. Cases with missing data for key variables (Ki-67, grade, VI, or survival outcome) were excluded from analysis.

## 3. Results

### 3.1. Patient baseline characteristics and follow-up

A total of 1243 patients were initially screened, and 846 were included, comprising 389 patients with breast cancer (46.0%) and 457 with colorectal cancer (54.0%). Follow-up was censored on June 30, 2025, consistent with the methods. The manuscript currently reports a median follow-up of 52.3 months (range, 3.2–112.5 months), 53 patients (6.3%) lost to follow-up, 187 deaths (22.1%), and 260 DFS events (30.7%). Baseline characteristics are presented in Table 1. Breast cancer patients were almost exclusively female (99.2%) and younger than colorectal cancer patients (55.2 ± 10.7 vs 63.4 ± 12.1 years, *P* < .001). The reported proportion classified as high Ki-67 was 53.5% in breast cancer and 69.4% in colorectal cancer. Luminal B was the most common reported breast subtype (42.9%), and pMMR predominated in colorectal cancer (76.6%).

**Table 1:** Comparison of baseline clinicopathological characteristics between breast and colorectal cancer patients.

Characteristic	Breast cancer (n = 389)	Colorectal cancer (n = 457)	Statistic	*P* value
Age (yr)	55.2 ± 10.7	63.4 ± 12.1	*t* = −11.245	<.001
Sex, n (%)			χ^2^ = 380.142	<.001
Male	3 (0.8)	249 (54.5)		
Female	386 (99.2)	208 (45.5)		
TNM stage, n (%)			*Z* = −3.891	<.001
Stage I	112 (28.8)	89 (19.5)		
Stage II	183 (47.0)	216 (47.3)		
Stage III	94 (24.2)	152 (33.3)		
Ki-67 index, n (%)			χ^2^ = 35.672	<.001
Low expression (<14%/20%)	181 (46.5)	140 (30.6)		
High expression (≥14%/20%)	208 (53.5)	317 (69.4)		
Pathological grade, n (%)			*Z* = −1.884	.060
Grade I/well-differentiated	87 (22.4)	102 (22.3)		
Grade II/moderately differentiated	215 (55.3)	274 (60.0)		
Grade III/poorly differentiated	87 (22.4)	81 (17.7)		
Vascular invasion, n (%)	86 (22.1)	124 (27.1)	χ^2^ = 2.889	.089
Molecular subtype/MSI status, n (%)				
Luminal A	135 (34.7)	–		
Luminal B	167 (42.9)	–		
HER2-overexpressing	52 (13.4)	–		
Triple-negative	35 (9.0)	–		
pMMR	–	350 (76.6)		
dMMR	–	107 (23.4)		
Perineural invasion, n (%)	45 (11.6)	118 (25.8)	χ^2^ = 28.541	<.001
Positive margin, n (%)	18 (4.6)	31 (6.8)	χ^2^ = 1.850	.174

Mann–Whitney *U* test; Ki-67 cutoff: 14% for breast cancer, 20% for colorectal cancer.

dMMR = deficient mismatch repair, HER2 = human epidermal growth factor receptor 2, pMMR = proficient mismatch repair, MSI = microsatellite instability, SD = standard deviation, TNM = tumor-node-metastasis.

*P* < .05.

### 3.2. Univariate analysis of prognostic factors

Findings from the univariate Cox regression assessing overall survival are detailed in Table 2. Within the entire cohort (n = 846), increased age (modeled as a continuous variable), advanced TNM stage (stage III vs stage I), elevated Ki-67 expression, high pathological grade (grade III/poor differentiation), and the presence of VI were each significantly associated with poorer survival outcomes. Notably, the prognostic influence of Ki-67 varied across breast cancer molecular subtypes. A significant association with survival was observed in luminal B (HR = 1.62, *P* = .029) and triple-negative (HR = 1.85, *P* = .042) cancers, but not in luminal A or HER2-overexpressing subtypes. Similarly, the prognostic strength of VI was context-dependent in colorectal cancer. It served as a potent risk factor in tumors with pMMR (HR = 2.34, *P* < .001). Conversely, for tumors with dMMR, VI showed a nonsignificant trend toward increased risk (HR = 1.48, *P* = .182).

**Table 2:** Univariate cox proportional hazards regression analysis for overall survival.

Variable	Group	No. of patients (events)	HR (95% CI)	*P* value
Overall analysis (n = 846)				
Age (per 10-yr increase)	Continuous	846 (187)	1.32 (1.16–1.50)	<.001
Sex	Female vs male	594 (120) vs 252 (67)	0.95 (0.70–1.28)	.723
Tumor type	Colorectal vs breast	457 (112) vs 389 (75)	1.21 (0.90–1.63)	.207
TNM stage	Stage II vs stage I	399 (91) vs 201 (28)	1.78 (1.16–2.72)	.008
	Stage III vs stage I	246 (68) vs 201 (28)	2.89 (1.87–4.48)	<.001
Ki-67 index	High vs low	525 (125) vs 321 (62)	1.52 (1.12–2.06)	.007
Pathological grade	Grade III/poor vs I/II	168 (52) vs 678 (135)	1.78 (1.29–2.45)	<.001
Vascular invasion	Present vs absent	210 (59) vs 636 (128)	1.64 (1.20–2.23)	.002
Perineural invasion	Present vs absent	163 (48) vs 683 (139)	1.45 (1.04–2.02)	.027
Breast cancer subgroup analysis				
Ki-67 (luminal A)	High vs low	50 (10) vs 85 (12)	1.49 (0.84–2.65)	.309
Ki-67 (luminal B)	High vs low	91 (28) vs 76 (15)	1.62 (1.05–2.50)	.029
Ki-67 (HER2-overexpressing)	High vs low	40 (11) vs 12 (3)	1.41 (0.65–3.05)	.380
Ki-67 (triple-negative)	High vs low	27 (10) vs 8 (2)	1.85 (1.02–3.34)	.042
Colorectal cancer subgroup analysis				
Vascular invasion (pMMR)	Present vs absent	70 (28) vs 280 (52)	2.34 (1.66–3.29)	<.001
Vascular invasion (dMMR)	Present vs absent	21 (5) vs 86 (14)	1.48 (0.83–2.65)	.182

CI = confidence interval, dMMR = deficient mismatch repair, HER2 = human epidermal growth factor receptor 2, HR = hazard ratio, pMMR = proficient mismatch repair, TNM = tumor-node-metastasis.

*P* < .05.

### 3.3. Multivariate analysis of independent prognostic factors

Variables with *P* < .1 in the univariate analysis (age, TNM stage, Ki-67, pathological grade, VI, and perineural invasion) were all entered into the multivariate Cox proportional hazards model. As shown in Table 3, after adjusting for other confounding factors, age (HR = 1.24, *P* = .002), TNM stage III (HR = 2.18, *P* < .001), Ki-67 high expression (HR = 1.38, *P* = .043), and VI (HR = 1.45, *P* = .026) remained independent risk factors for overall survival. However, the independent prognostic significance of pathological grade disappeared (grade III/poorly differentiated vs grades I/II: HR = 1.31, *P* = .062), and perineural invasion also became nonsignificant (*P* = .112).

**Table 3:** Multivariate cox proportional hazards regression analysis for overall survival (n = 846).

Variable	Reference group	β value	Wald χ^2^	Adjusted HR (95% CI)	*P* value
Age (per 10-yr increase)	–	0.215	9.624	1.24 (1.08–1.42)	.002
TNM stage			22.415		<.001
Stage II	Stage I	0.324	2.789	1.38 (0.96–1.99)	.095
Stage III	Stage I	0.779	19.223	2.18 (1.57–3.03)	<.001
Ki-67 index	Low expression	0.319	4.096	1.38 (1.01–1.88)	.043
Pathological grade	Grades I/II	0.270	3.478	1.31 (0.99–1.74)	.062
Vascular invasion	Absent	0.373	4.956	1.45 (1.05–2.01)	.026
Perineural invasion	Absent	0.230	2.531	1.26 (0.95–1.67)	.112

Tumor type (breast/colorectal) was also included in the model as a covariate; its *P* > .05 and is not listed separately in the table.

CI = confidence interval, HR = hazard ratio, TNM = tumor-node-metastasis.

### 3.4. Exploration of a multi-indicator combined prognostic score

To illustrate the cumulative prognostic information contained in the 2 dichotomous variables that retained independent statistical significance in the original multivariable analysis (high Ki-67 expression and VI), an exploratory 0 to 2 point score was constructed, assigning 1 point for each factor present. As shown in Table 4, the risk of death increased with higher scores (trend test *P* < .001), and patients with a score of 2 had a 3.19-fold higher risk of death than those with a score of 0. This score was derived and evaluated in the same retrospective cohort and, therefore, should not be interpreted as a validated clinical prediction instrument.

**Table 4:** Relationship between the combined score based on Ki-67 and vascular invasion and patient prognosis.

Risk score	Risk factor combination	No. of patients (n)	No. of death events (n)	5-year OS rate (%)	HR (95% CI)	*P* value
0	Ki-67 low and VI (−)	268	38	88.5	1.00 (reference)	–
1	Ki-67 high or VI (+)	438	98	77.3	1.92 (1.31–2.80)	.001
2	Ki-67 high and VI (+)	140	51	62.1	3.19 (2.08–4.88)	<.001

HR is adjusted for age and TNM stage, with the 0-score group as reference. Trend test: χ^2^ = 36.728, *P *< .001.

CI = confidence interval, HR = hazard ratio, OS = overall survival, TNM = tumor-node-metastasis, VI = vascular invasion.

### 3.5. Survival curves

**Figure 1.**
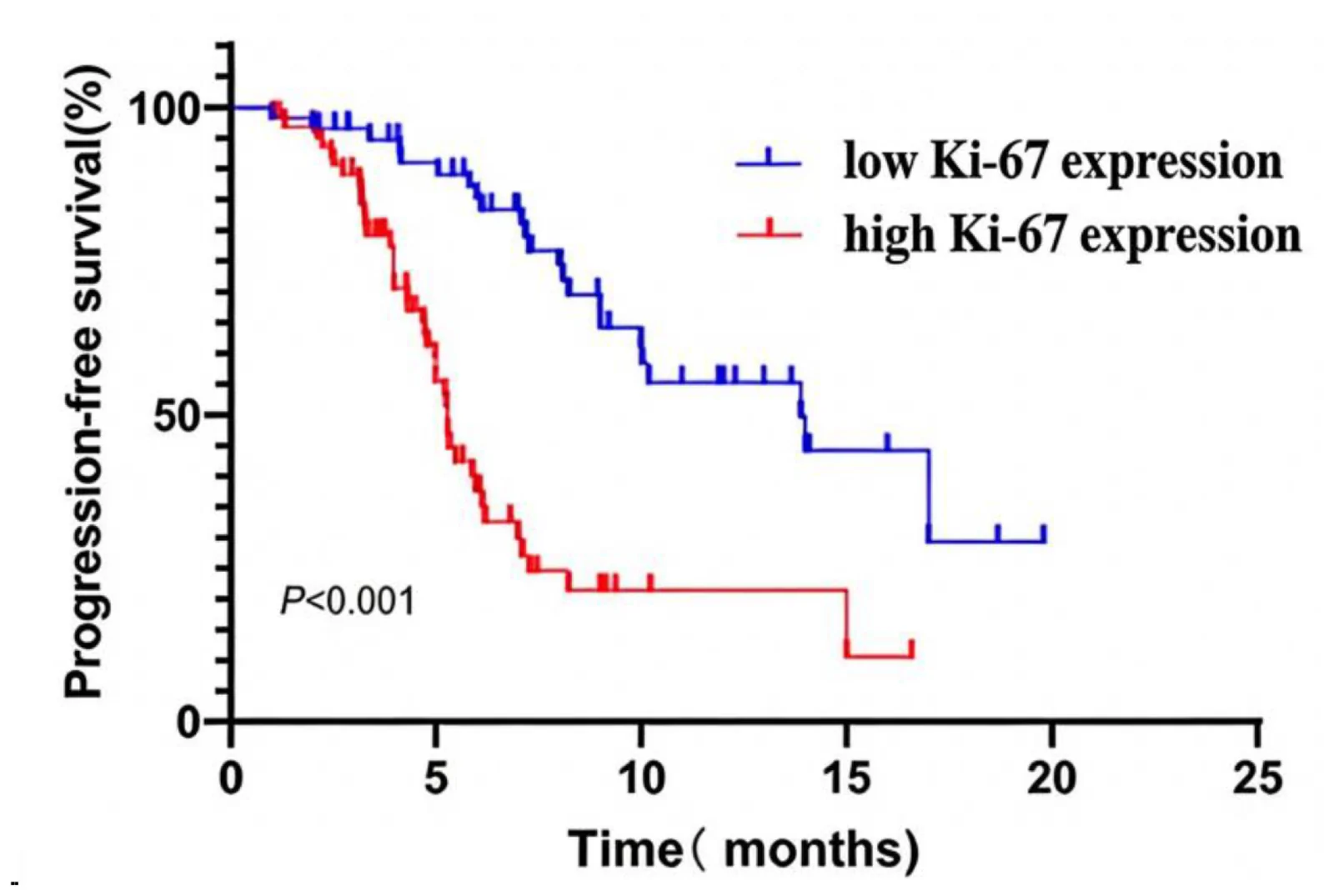
Kaplan–Meier curves for overall survival (OS) stratified by Ki-67 expression status in the entire cohort. Patients with high Ki-67 expression showed significantly worse OS compared to those with low Ki-67 expression (Log-rank test, *P *= .04).

**Figure 2.**
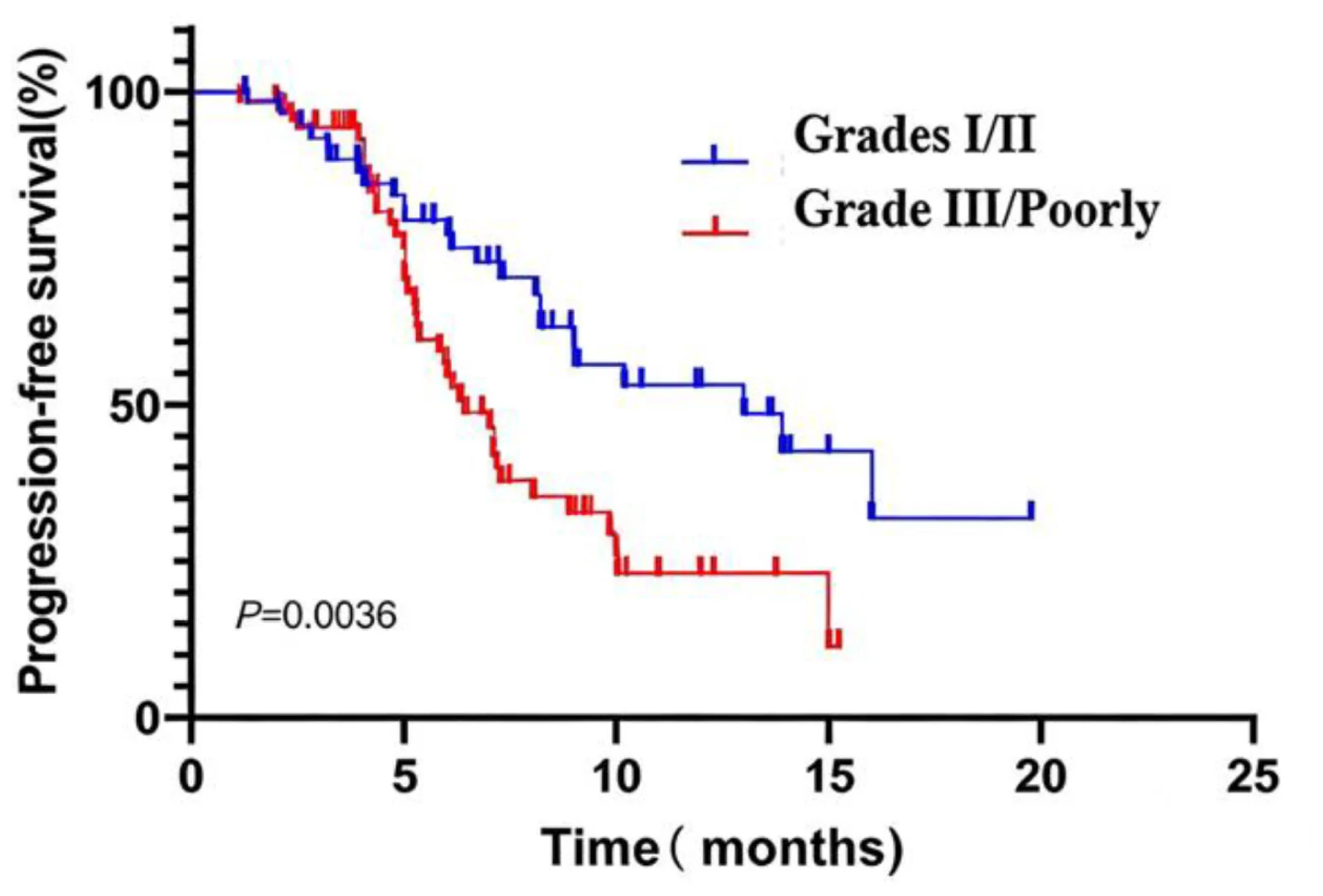
Kaplan–Meier curves for overall survival stratified by pathological grade.

Kaplan–Meier survival curves demonstrated prognostic stratification capability. Figure 1 shows that the overall survival rate was significantly lower in the Ki-67 high expression group compared to the low expression group (log-rank *P* = .04). Figure 2 shows that the disease-free survival rate was significantly worse in the VI-positive group (log-rank *P* < .001). In contrast, the survival curves stratified by pathological grade (grades I/II vs grade III/poorly differentiated), although significantly different before multivariate adjustment (univariate *P* < .001), showed limited independent contribution after correcting for other factors, with the curves crossing in the later period.

### 3.6. Exploratory discrimination assessed by ROC curves

**Figure 3.**
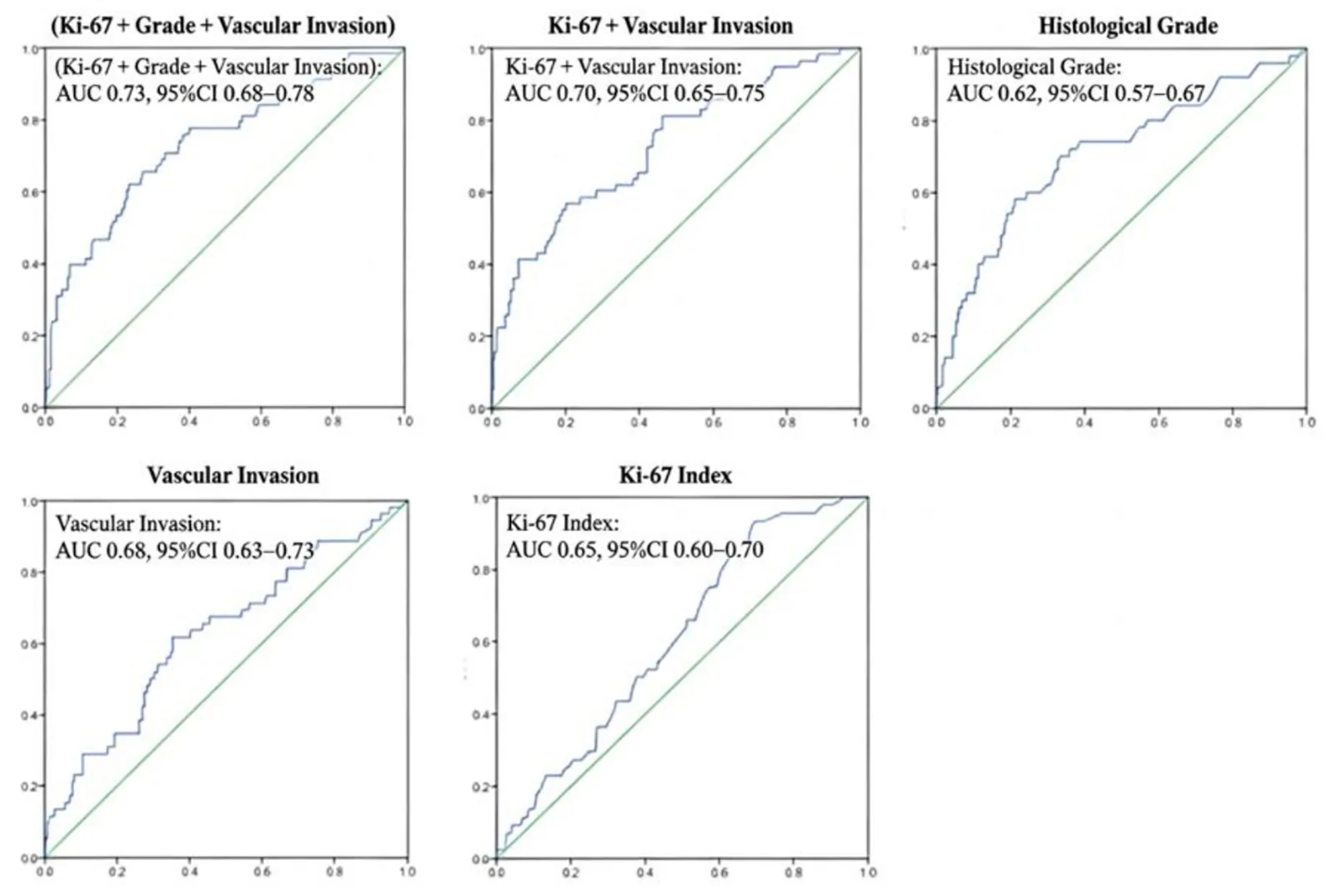
ROC curves for predicting 5-year overall survival. The figure compares the ROC curves of individual pathological indicators (Ki-67, histological grade, vascular invasion) with 2 combined models: the simple risk score (0–2 points) and the probability derived from a multivariable logistic regression model. AUC = area under the curve, CI = confidence interval, ROC = receiver operating characteristic.

As specified in the analysis, ROC curves were used to provide an exploratory comparison of apparent 5-year discrimination among the individual pathological indicators and combined models. The AUC values are presented in Table 5. Ki-67 showed an AUC of 0.65 (95% CI: 0.60–0.70), histological grade an AUC of 0.62 (95% CI: 0.57–0.67), and VI an AUC of 0.68 (95% CI: 0.63–0.73). The simple Ki-67/VI score had an AUC of 0.70 (95% CI: 0.65–0.75), while the multivariable logistic regression model incorporating Ki-67, grade, and VI had an AUC of 0.73 (95% CI: 0.68–0.78). DeLong test showed higher AUCs for the combined model than for the single indicators (all *P* < .05), as previously reported. Because these analyses were performed in the same cohort in which the models were constructed and were not supplemented by bootstrap correction, time-dependent AUC, calibration, or external validation, these results represent apparent discrimination only and do not establish clinical utility (Figure 3).

**Table 5:** Discriminatory performance of individual and combined pathological indicators for predicting 5-year overall survival.

Prognostic model/indicator	Area under the curve (AUC)	95% confidence interval (CI)	*P* value
Ki-67 index	0.65	0.60–0.70	<.001
Histological grade	0.62	0.57–0.67	<.001
Vascular invasion	0.68	0.63–0.73	<.001
(Ki-67 + vascular invasion)	0.70	0.65–0.75	<.001
(Ki-67 + grade + vascular invasion)	0.73	0.68–0.78	<.001

## 4. Discussion

This retrospective study explored the prognostic associations of Ki-67, histological grade, and VI in 846 patients with breast or colorectal cancer. The pooled cohort was used as an exploratory cross-cancer framework because these variables are routinely reported in both malignancies; it was not intended to imply biological equivalence between the 2 cancer types or to create a universal prognostic model. The principal interpretation therefore rests on the cancer-specific and subtype-specific findings. In the original multivariable analysis, high Ki-67 expression and VI remained associated with poorer overall survival, whereas histological grade did not retain independent statistical significance. The observed subgroup patterns suggested that the association of Ki-67 was more evident in luminal B and triple-negative breast cancer and that the association of VI appeared stronger in pMMR than in dMMR colorectal cancer. These subgroup observations should be interpreted as exploratory rather than definitive evidence of effect modification.

After adjusting for confounders such as TNM stage and age, high Ki-67 expression and VI remained independent predictors of poor patient prognosis. Ki-67, a key cell cycle protein, directly quantifies the proliferative activity of tumor cells through its expression level.^[10,11]^ The findings of this study are consistent with conclusions from multiple solid tumor studies. Zhao et al^[12]^ found in a study of clear cell renal cell carcinoma with tumor thrombus that high Ki-67 expression (cutoff 30%) was significantly associated with worse recurrence and cancer-specific survival and closely linked with aggressive pathological features. In rectal neuroendocrine tumors, the study by Sugimoto et al^[13]^ also confirmed that a Ki-67 labeling index >3.0% is an independent risk factor for predicting metastasis. The present study indicates that high proliferative activity is a common adverse biological feature across both breast and colorectal cancer. VI, serving as morphological evidence of hematogenous or lymphatic dissemination of tumor cells, had its independent prognostic value confirmed in this study. This finding is supported by extensive literature. A large review by Mirza et al^[14]^ on node-negative breast cancer noted that VI is one of the significant predictors of survival outcome. The systematic review by Knijn et al^[15]^ established the prognostic significance of intramural VI in colorectal cancer. This study further consolidates the status of VI as a fundamental yet crucial prognostic marker through a large sample dataset.

Pathological grade (Nottingham grade/differentiation degree) lost its independent prognostic significance in the multivariate analysis. This suggests that the prognostic information contained within pathological grade may be overlapped or substituted by indicators with more objective quantitative properties or reflecting different biological dimensions, such as TNM stage and the Ki-67 index.^[16]^ High-grade tumors are often accompanied by higher proliferative activity and a more advanced stage.^[17]^ Research by Ahmed et al^[7]^ also indicated that in colorectal cancer, the Ki-67 index requires comprehensive assessment alongside other factors for its prognostic value. This finding prompts reflection that in the era of molecular pathology, relying solely on morphological grading for prognosis judgment has limitations and necessitates integrated evaluation with indices such as the proliferation index.

The breast cancer subgroup findings also require cautious interpretation. The association of Ki-67 appeared more evident in luminal B and triple-negative tumors than in luminal A tumors. However, Ki-67 can contribute to surrogate luminal classification in routine breast pathology, and the detailed historical rules used to separate luminal A from luminal B were not uniformly recoverable from all archived reports in this retrospective cohort. This creates a potential incorporation-bias concern when interpreting Ki-67 within luminal subgroups. The present findings should therefore be viewed as hypothesis-generating observations about biological context rather than as evidence that Ki-67 alone should determine treatment intensity. The study by Viale et al^[18]^ illustrates the close relationship between Ki-67 assessment and luminal classification, while Sandilya et al^[6]^ reported an association between Ki-67 and histological grade. Prospective studies using explicitly prespecified and reproducible subtype definitions are needed to clarify the independent prognostic contribution of Ki-67 within each breast cancer subtype.^[15]^

In colorectal cancer, VI showed a stronger prognostic association in the pMMR subgroup than in the dMMR subgroup in the analyses reported here. dMMR colorectal cancer has distinct biological and immune characteristics, which may partly contribute to differences in observed prognostic patterns. However, the absence of statistical significance in a smaller subgroup should not be interpreted as proof of absence of an effect, and the present retrospective analysis does not establish a treatment-predictive role for VI according to MMR status. The findings therefore support further study of MMR status as a biological context for interpreting conventional pathological markers, rather than direct treatment recommendations based on VI alone.

Based on the independent prognostic value of Ki-67 and VI, the simple combined scoring model constructed in this study showed that the risk of death increased stepwise and significantly with the accumulation of risk factors. This integration approach aims to overcome the limitations of single indicators and provide more robust risk stratification. This concept aligns with several studies. Kubik et al^[19]^ noted in a meningioma study that combining the Ki-67 index and CD34 microvessel density could more effectively predict recurrence risk and functional recovery potential. Mestrum et al^[20]^ found in soft tissue sarcoma that the Ki-67 index, in conjunction with features such as tumor size, grade, necrosis, and VI, synergistically influenced metastasis risk. Our ROC curve analysis provides a complementary, quantitative perspective on the discriminatory power of these markers. While univariate and multivariate Cox regressions established the independent prognostic value of Ki-67 and VI, the ROC analysis quantified their ability to distinguish between patients who did and did not survive to 5 years. The finding that the combined models – particularly the logistic regression model integrating all 3 markers – achieved a significantly higher AUC (0.73) than any single indicator reinforces the conclusion derived from the survival analyses: integration of multiple pathological parameters enhances prognostic stratification. Importantly, the simple clinical risk score (AUC = 0.70) performed nearly as well as the more complex statistical model, underscoring its practical utility. This synergy between the time-to-event analysis (Cox model) and binary classification accuracy (ROC analysis) strengthens the rationale for moving beyond single-marker interpretation towards an integrated, context-aware assessment framework in clinical pathology reporting.^[21]^

The 0 to 2 point Ki-67/VI score was intentionally simple and based on variables available in routine pathology reports. Nevertheless, simplicity alone does not establish clinical usefulness. In response to the reviewer’s concern, we have removed wording implying clinical utility, clinical dissemination, or readiness for treatment decision-making. Formal evaluation of a prognostic model would require prespecified model development, internal validation, calibration assessment, evaluation of time-dependent discrimination, and independent external validation. Future studies may determine whether this exploratory combination provides reproducible incremental prognostic information beyond established clinicopathological factors.

The innovativeness of this study is mainly reflected in 2 aspects: firstly, through cross-cancer comparison, it revealed the universality of Ki-67 and VI as prognostic factors; secondly, by delving into the molecular subtype level, it uncovered the conditional dependency of these traditional indicators’ prognostic value, particularly the attenuation of VI’s predictive power in dMMR colorectal cancer, providing new evidence for understanding how tumor biological heterogeneity influences the interpretation of morphological indicators.

This study has several limitations. First, its single-center retrospective design is subject to selection bias and limits generalizability. Second, breast and colorectal cancer differ substantially in baseline prognosis, biology, and treatment; therefore, the pooled analysis should be interpreted only as an exploratory framework, with greater emphasis placed on cancer-specific findings. Third, although postoperative treatment information was collected, treatment regimens and completion were heterogeneous and were not comprehensively adjusted in the original prognostic models. Treatment allocation is also strongly influenced by stage and tumor biology, creating potential confounding by indication; consequently, the observed associations should be interpreted as prognostic associations rather than treatment-independent causal effects. Fourth, the detailed historical criteria distinguishing luminal A from luminal B were not uniformly recoverable from all archived reports, and the possible involvement of Ki-67 in surrogate luminal classification creates potential incorporation bias. Fifth, Ki-67 was analyzed using predefined categorical thresholds for the main survival analyses. We did not add post hoc RCS modeling in the present revision; therefore, information loss due to dichotomization and potential nonlinear associations remain unresolved. Sixth, the exploratory Ki-67/VI score was derived and assessed in the same cohort without bootstrap correction, time-dependent AUC, calibration, decision-curve analysis, or external validation and should not be regarded as a validated clinical tool. Finally, the median follow-up of 52.3 months may be insufficient to capture some late events, particularly in more indolent breast cancer subtypes.

Future research directions should include: conducting multicenter prospective studies to validate the findings of this research; utilizing digital pathology and artificial intelligence technologies for more precise and reproducible quantitative analysis of the Ki-67 index and VI; and further exploring whether, within specific subtypes, there exist molecular or immune markers with greater predictive value beyond Ki-67 and VI, to construct a next-generation prognostic assessment system integrating macroscopic morphology and microscopic molecules.

## 5. Conclusion

In this single-center retrospective cohort, high Ki-67 expression and VI were associated with poorer overall survival after adjustment in the original multivariable analysis, whereas histological grade did not retain independent statistical significance. The observed subgroup patterns suggest that the prognostic associations of these conventional pathological indicators may vary across tumor biological contexts, including breast cancer subtype and colorectal cancer MMR status. The Ki-67/VI score provides an exploratory illustration of cumulative risk stratification but is not a validated clinical prediction tool. These findings should be confirmed in independent cohorts using prespecified subtype definitions, more comprehensive treatment adjustment, continuous-variable analyses, and formal internal and external validation before any clinical application is considered.

## Author contributions

**Conceptualization:** Hongyan Ji, Binzheng Liu, Lisha Xie.

**Data curation:** Hongyan Ji, Binzheng Liu, Lisha Xie.

**Formal analysis:** Hongyan Ji, Binzheng Liu, Lisha Xie.

**Funding acquisition:** Hongyan Ji.

**Investigation:** Hongyan Ji.

**Writing – original draft:** Hongyan Ji.

**Writing – review & editing:** Hongyan Ji.
